# Effect of Annealing Process and Molecular Weight on the Polymorphic Transformation from Form II to Form I of Poly(1-butene)

**DOI:** 10.3390/polym15040800

**Published:** 2023-02-05

**Authors:** Zhenkang Zhang, Yanhu Xue, Rui Li, Wei Liu, Peng Liu, Xiangling Ji

**Affiliations:** 1 College of Chemical Engineering and Materials Science, Tianjin University of Science and Technology, Tianjin 300457, China; 2 State Key Laboratory of Polymer Physics and Chemistry, Changchun Institute of Applied Chemistry, Chinese Academy of Sciences, Changchun 130022, China

**Keywords:** poly(1-butene), molecular weight, crystal transformation, annealing process

## Abstract

Poly(1-butene) (PB-1) resin has excellent mechanical properties, outstanding creep resistance, environmental stress crack resistance and other excellent properties. However, PB-1 resin experiences a crystal transformation for a period, which seriously affects the production efficiency and directly restricts its large-scale commercial production and application. The factors affecting the crystal transformation of PB-1 are mainly divided into external and internal factors. External factors include crystallization temperature, thermal history, nucleating agent, pressure, solvent induction, etc., and internal factors include chain length, copolymerization composition, isotacticity, its distribution, etc. In this study, to avoid the interference of molecular weight distribution on crystallization behavior, five PB-1 samples with narrow molecular weight distribution (between 1.09 and 1.44) and different molecular weights (from 23 to 710 k) were chosen to research the influence of temperature and time in the step-by-step annealing process and molecular weight on the crystal transformation by differential scanning calorimetry (DSC). When the total annealing time was the same, the step-by-step annealing process can significantly accelerate the rate of transformation from crystal form II to I. PB-1 samples with different molecular weights have the same dependence on annealing temperature, and the optimal nucleation temperature (i.e., low annealing temperature, T_l_) and growth temperature (i.e., high annealing temperature, T_h_) were −10 °C and 40 °C, respectively. At these two temperatures, the crystal form I obtained by step-by-step annealing had the highest content; other lower or higher annealing temperatures would reduce the rate of crystal transformation. When the annealing temperature was the same, crystal form I first increased with annealing time t_l_, then gradually reached a plateau, but the time to reach a plateau was different. The crystalline form I contents of the samples with lower molecular weight increased linearly with annealing time t_h_. However, the crystalline form I contents of the samples with higher molecular weight increased rapidly with annealing time t_h_ at the beginning, and then transformation speed from form II to form I slowed down, which implied that controlling T_l_/t_l_ and T_h_/t_h_ can tune the different contents of form I and form II. At the same T_l_/t_l_ or T_h_/t_h_, with increasing molecular weight, the transformation speed from form II to form I via the step-by-step annealing process firstly increased and then slowed down due to the competition of the number of linked molecules and molecular chain mobility during crystallization. This study definitely provides an effective method for accelerating the transformation of poly(1-butene) crystal form, which not only has important academic significance, but also has vital industrial application.

## 1. Introduction

Poly(1-butene) (PB-1) resin was first synthesized by Natta in 1954 using a Ziegler-Natta catalyst [1]. Because PB-1 has excellent mechanical properties, outstanding creep resistance, environmental stress crack resistance and other excellent properties, it is widely used in many fields, such as geothermal pipes, easy-tear films and food packaging, and has the reputation of “plastic gold” [2]. Compared with other general polymers (such as polyethylene, polypropylene, etc.), PB-1 is a polymorphic polymer due to its special crystallization behavior, and the commercial value of PB-1 resin is less recognized.

PB-1 resin has four kinds of crystal forms: I, I’, II and III [3,4,5]. PB-1 can form different crystal forms under different crystallization conditions, the most vital ones are crystal forms I and II. Generally, PB-1 resin cooled from the melt cannot directly obtain the thermodynamically stable crystal form I, but firstly forms crystal form II due to kinetic advantages. PB-1 crystal form II is a metastable state, and it can spontaneously transform into stable crystal form I [6,7,8,9]. Owing to different crystal structures, from II to I phase transformation will make the sample shrink and then increase the density, and this shrinkage will lead to deformation of the sample during use [10]. Therefore, the as-prepared sample cannot be used immediately; it needs to keep some days until phase transition is completed, which is a challenge for practical production application [11].

Researchers have studied the influence of internal factors (including molecular weight, co-monomer content, isotacticity, etc.) and external factors (crystallization temperature, crystallization time, stress, pressure, etc.) on crystal transformation. Sun et al. investigated the influence of lamella thickness from II to I crystal transition and found that the transition from thick lamellae was always better than thin lamellae [12]. It has also been found that a certain content of crystal form II can promote the progress of from II to I phase transition [13]. Chen et al. utilized poly(1-butene) with different ethylene co-unit contents and found that ethylene co-monomer inhibited crystallization and significantly accelerated the spontaneous II-to-I transition [14]. He et al. found that the crystallinity and lamellar thickness of crystal form I of poly(1-butene) containing a short isotactic sequence were directly proportional to temperature [15]. Some researchers have discussed some methods that can speed up from II to I phase transformation, such as the introduction of co-monomers [14,16], stress effects [17], pressurized CO_2_ [18,19], solvent treatment [20,21], and heat treatment [22,23,24]. Miyoshi et al. used PB-1 with low regularity and found that these samples can be directly crystallized into crystal form II. However, the lamellae at this time were very thin, and high mechanical strength was not obtained [8]. The crystallization kinetics of PB-1 resin was studied by Men et al. using Tammann’s two-stage nucleation method [22,24]. Under certain annealing conditions, the transformation speed of the higher molecular weight samples increased with the crystallization temperature, while the lower molecular weight samples was negatively correlated with the crystallization temperature [24]. However, the study by Men et al. used a commercial PB-1 resin with a very complex chain structure, which possibly would affect the final results. Under different nucleation temperature and growth temperature, the influence of narrow distribution poly(1-butene) samples of different molecular weights on the crystal transformation is not entirely clear.

Xue et al. designed a new preparative temperature rising elution fractionation (P-TREF) equipment with a wide temperature range, which could separate PB-1 successfully according to the crystallization ability [25]. Xue et al. also separated PB-1 according to molecular weight using the solvent gradient fractionation (SGF) method, and the weight average molecular weight of the fractions increased from 31.9 to 1217.4 k with the content of good solvent from 0 to 100 vol% [26]. The chain structure of PB-1 resin obtained in industrial production is usually complex, the results obtained from original resins often present only average values, which are insufficient to study the relationship between chain microstructure and property [25,26,27]. The molecular weight of PB-1 resin and its distribution, isotacticity, and co-monomer content all affect its crystallization behavior; it was difficult to clearly understand the influence of a certain factor on its crystallization behavior just using the original sample. Therefore, it was necessary to classify resins according to certain principles to obtain narrow distribution fractions with similar chain structures, which is of great significance for studying the crystallization kinetics of PB-1 [25]. Table 1 summarizes the information of the PB samples used by the predecessors, and it can be observed that the samples usually have a broad molecular weight distribution. 

In order to avoid the interference of molecular weight distribution on crystallization behavior, herein, five narrow distribution PB-1 samples with different molecular weights (from 23 to 710 k) were selected to research the influence of annealing condition and molecular weight on the crystal transformation systematically.

## 2. Experimental

### 2.1. Materials

The poly(1-butene) homopolymer resin coded PB0110 by LyondellBasell Corporation was fractionated according to molecular weight. Here, the method of solvent gradient fractionation (SGF) was used by Xue et al. as reported in literature [26]. First, the sample was dissolved in TMB/BCS mixed solvent, and then the solution was transferred to a fractionation column, the temperature was reduced from 150 °C to room temperature of 25 °C, and after a period of equilibrium, it was eluted with poor solvent, and finally the temperature was raised to 110 °C. At the same elution temperature (110 °C), fractions with different molecular weights were obtained by changing the ratio of good solvent to poor solvent. Five fractions with different molecular weights were selected as samples in the study, which were all narrow molecular weight distribution, and were listed in Table 2. 

### 2.2. Differential Scanning Calorimetry (DSC)

The DSC test was conducted on the Q100 differential scanning calorimeter produced by TA Company. About 6 mg of the sample was weighed for DSC test. The different test procedures were described as follows. 

Method 1. The entire test process was carried out under nitrogen atmosphere. Firstly, in order to eliminate the thermal history, the temperature was raised to 200 °C for 5 min, then the crystallization information of the sample was obtained by cooling to 0 °C for 5 min, increasing to 200 °C again with the rate of 10 K/min. The cooling curve and the second heating curve were its crystallization and melting curve, respectively. 

**Scheme 1:**
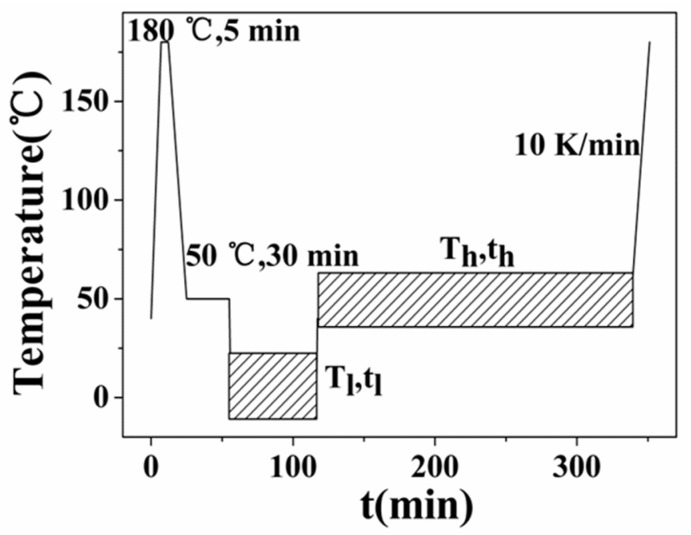
Schematic illustration of different annealing conditions for PB-1 samples.

Method 2. The annealing process was shown in Scheme 1 and carried out under nitrogen atmosphere. First, the prepared sample was heated to 180 °C for 5 min. Then, it was cooled to 50 °C and held for 30 min for the complete crystallization of form II, then it was cooled to different low annealing temperature (T_1_) for different time (t_l_), and then raised to different high annealing temperature (T_h_) for different time (t_h_). Finally, the annealed sample was heated to 180 °C to obtain the melting curve, from which the contents of forms II and I were obtained via melting peak fitting and integrating procedure considering exponentially modified Gaussian functions. The melting ranges of forms II and I are around 115–120 and 125–130 °C, respectively.

**Figure 1:**
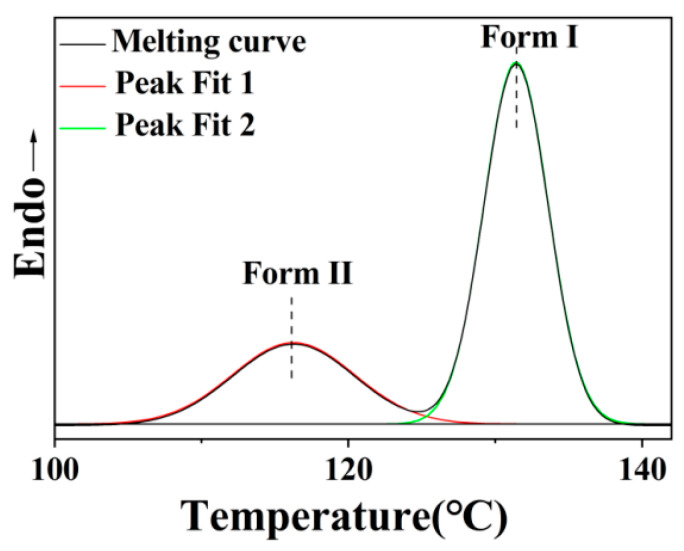
Example of peak fits for crystal forms I and II from the DSC melting curve (Sample F2).

Owing to the different crystal structures of different crystal forms, the positions of the melting peaks would also be different. The melting enthalpy of crystal forms II and I were received through sub-peak fitting and integration of the melting curve as was shown in Figure 1 [29]. PeakFit software was used to fit the melting curve. Firstly, the data were imported into the software, and then the temperature range and baseline were selected. The peak value of the fitting curve corresponded to the peak value of the melting curve represented by crystal forms II and I, and the fitting curve with exponentially modified Gaussian functions was adjusted to coincide with the melting curve as much as possible. Finally, the melting peak areas of crystal forms II and I were obtained. The crystal form transformation ratio could be expressed through the content of crystal form I obtained by Formula (1).

(1) $$X_{I}=\frac{A_{I}/\Delta H_{\mathrm{id},I}}{A_{I}/\Delta H_{\mathrm{id},I}+{\;A}_{\mathrm{II}}/\Delta H_{\mathrm{id},\mathrm{II}}}$$

In the formula, A_I_ and A_II_ are the melting peak areas of crystal form I and II, respectively, ΔH_id,I_ and ΔH_id,II_ are the melting enthalpies of the ideal crystals when crystal forms I and II are completely crystallized, ΔH_id,I_ and ΔH_id,II_ are 141 and 62 J/g, respectively [8].

## 3. Results and Discussion

### 3.1. Characterization of Samples

**Figure 2:**
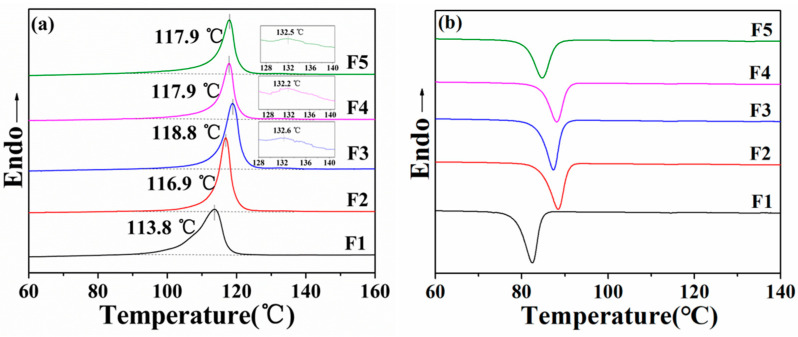
DSC heating (**a**) and cooling curves (**b**) of five samples (F1–F5).

Figure 2 shows DSC results of five samples (F1–F5) with different molecular weights by the DSC method 1. The detailed data, such as melting temperature and melting enthalpy, were recorded in Table 3. It was observed that PB-1 samples F1 (23 k) and F2 (109 k) with lower molecular weights only had one melting peak at 113.8 and 116.9 °C, respectively; the samples F3 (201 k), F4 (360 k), and F5 (710 k) with higher molecular weights had two melting peaks at 118.8 °C/132.6 °C, 117.9 °C/132.2 °C, and 117.9 °C/132.5 °C, respectively. Sample F1 had a slightly broad melting peak, and its melting enthalpy was 44.2 J/k, which was the greatest of the five samples. Because its molecular weight was the lowest of all the samples, its molecular chain had greater mobility and generated more crystal form II from the melt. It is well known that PB-1 resin was cooled from the melt firstly to form metastable form II crystals with kinetic advantages, and the crystal form II had a lower melting point; after that, it can become the thermodynamically stable form I along with a higher melting point. Therefore, samples F1 (23 k) and F2 (109 k) only contain the crystal form II in the heating curves, while the samples F3 (201 k), F4 (360 k), and F5 (710 k) had both crystal form II and form I corresponding to lower and higher melting peaks, respectively. These results imply that under the same annealing conditions, the higher molecular weight samples transformed from crystal form II to I faster than those with lower molecular weights. The five samples with different molecular weights only had one crystallization peak, and the characterization peaks were located at 82.4, 88.4, 87.4, 88.1, and 84.7 °C, respectively.

### 3.2. Influence of Stepwise Annealing and Molecular Weight on Crystal Transformation

**Figure 3:**
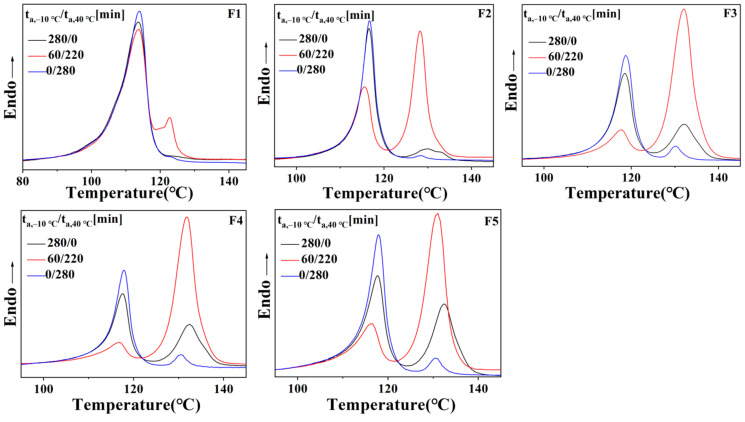
DSC melting curves of samples annealed under different stepwise annealing conditions.

**Figure 4:**
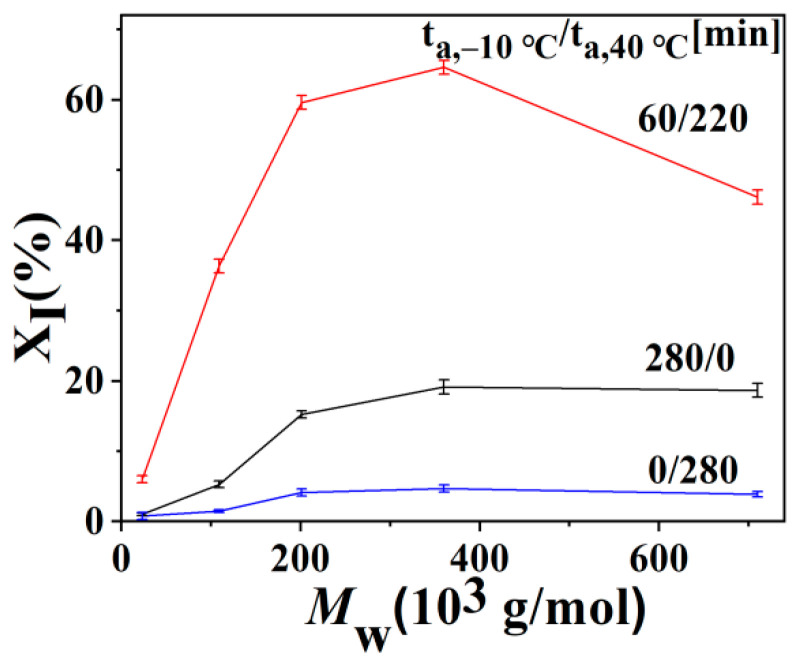
Variation of the crystal form I content (X_I_) of samples with molecular weight under different stepwise annealing conditions.

Five samples with different molecular weights (F1–F5) were also characterized by DSC method 2, first isothermally crystallized to form a kinetically dominant metastable crystal form II, and then stepwise annealing at −10 °C/40 °C or annealed at one temperature for the same time. During the annealing process, the form II would transform to form I, and finally the temperature was increased to 180 °C. Figure 3 was the DSC result obtained by the final heating curve after the annealing process, and the content of crystal form I was recorded in Table S1 (Supporting Information). Figure 4 shows the plot of form I content (X_I_) versus molecular weight (*M*_w_) under different stepwise annealing conditions. This could be observed in Figure 3 that the peak area of crystal form I obtained by the five samples annealed only at −10 °C (t_a, −10°C_/t_a, 40°C_ = 280/0) or 40 °C (t_a, −10°C_/t_a, 40°C_ =0/280) was very small, while the melting peak area of crystal form I obtained by stepwise annealing (t_a, −10°C_/t_a, 40°C_ = 60/220) increases significantly. This was also consistent with the previous conclusions obtained by Men et al., who found that the area of crystal form I obtained by annealing only at −16 °C (t_a, −16°C_/t_a, 20°C_ = 100/0) or 20 °C (t_a, −16°C_/t_a, 20°C_ = 0/100) was very small, while the melting peak area of form I obtained by stepwise annealing (t_a, −16°C_/t_a, 20°C_ = 30/70) increased significantly [22].

In Figure 4, the form I content X_I_ of the F1 (23 k) sample was only 1.02% when annealed at −10 °C and 0.78% when annealed at 40 °C, while it increased to 6.03% at −10 °C/40 °C stepwise annealing process. The form I content X_I_ of the F3 (201 k) sample was 15.23% when annealed at −10 °C and 4.12% when annealed at 40 °C, and it increased to 59.62% when annealed at −10 °C/40 °C step-by-step annealing. The form I content of the F5(710 k) sample was 46.15% when stepwise annealed at −10 °C/40 °C, significantly more than those only annealed at −10 °C (18.66%) or annealed at 40 °C (3.88%). Similar conclusions could be drawn for other samples. Compared with annealing at only one temperature, the form I content was more obtained by stepwise annealing, which accelerated the progress of crystal transformation. The low annealing temperature of the sample at −10 °C provided a greater subcooled temperature for the nucleation of crystal form I, and internal stress was generated during the cooling from 50 to −10 °C; it could provide a larger thermodynamic driving force to make it easier to overcome the energy barrier, thereby making the nucleation of form I easier, but when annealed at a low temperature, the molecular chain mobility was limited, and the nuclei cannot grow into crystals. When the poly(1-butene) sample was annealed at 40 °C, the molecular chain had a certain mobility, which facilitated the growth of crystal nuclei; however, it was not conducive to nucleation at a high temperature. The method of stepwise annealing was adopted, which involved annealing at a low temperature of −10 °C to promote form I nucleation and further annealing at 40 °C. The resulting crystal nuclei grew rapidly, thereby accelerating the transition from crystal form II to form I. 

With the increase in molecular weight from 23 to 710 k, the form I content X_I_ of sample annealed at −10 °C gradually increased from 1.02% to 5.28%, 15.23%, 19.14% and 18.66%, the form I content X_I_ of sample annealed at 40 °C gradually changed from 0.78% to 1.48%, 4.12%, 4.67% and 3.88%, the form I content X_I_ of sample with step-by-step annealed at −10 °C/40 °C gradually changed from 6.03% to 36.34%, 59.62%, 64.61% and 46.15%. It was obvious that with the increase in molecular weight from 23 to 710 k, the form I content X_I_ of the sample by step-by-step annealing at −10 °C or 40 °C firstly showed an increasing trend and then decreased. When the *M*_w_ is 360 k, the form I content X_I_ of the sample was the most in five samples under the same annealing time and temperature condition. When the molecular weight of poly(1-butene) sample increased from 23 k (F1) to 360 k (F4), the entanglement among molecular chains increased, which could transmit more internal stress, facilitate nucleation, and accelerate the progress of nucleation. However, the molecular weight continued to increase, and the chain mobility began to play an important role in the crystal transformation when there were enough intercrystalline links for nucleation. Molecular weight has double influence on the polymorphic transition from form II to form I [26]. High molecular weight samples also restricted chain mobility. Therefore, the form I content of the poly(1-butene) sample firstly showed an increasing trend and then decreased. The crystallization behavior of PB-1 could be adjusted by the low or high annealing temperature or time (T_l_/t_l_ or T_h_/t_h_).

### 3.3. Influence of Low Annealing Temperature T_l_ and Molecular Weight on Crystal Transformation

**Figure 5:**
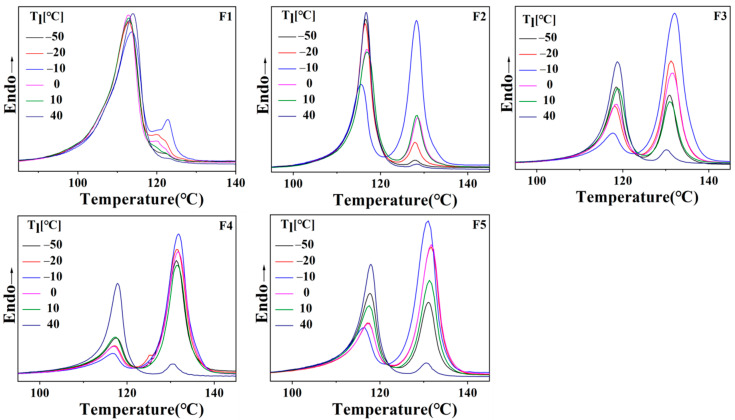
DSC melting curves of PB-1 samples annealed at different low annealing temperatures (T_1_).

**Figure 6:**
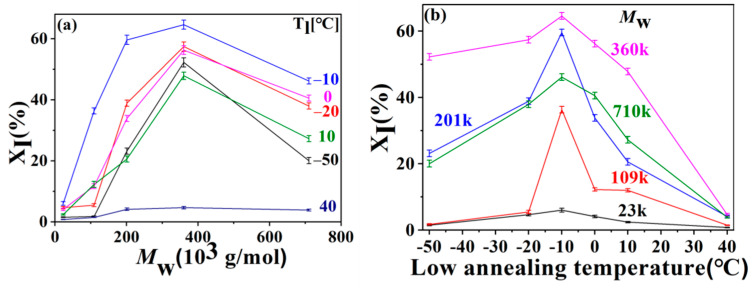
Variation of the form I content (X_I_) of samples with different molecular weights (**a**) or different low annealing temperatures (T_1_) (**b**).

After isothermal crystallization, the five samples (F1-F5) were annealed at different low annealing temperatures T_l_ (−50, −20, −10, 0, 10, 40 °C) for 60 min, then annealed at 40 °C (T_h_) for 220 min, finally increasing to 180 °C, and Figure 5 was the DSC result obtained by the final heating curve after heat treatment. The form I content X_I_ of the sample was recorded in Table S2. Figure 6 shows the plots of X_I_ versus *M*_w_ or T_1_. 

As shown in Figure 6a, when T_l_ was at −10 °C, the form I contents of five samples were all the largest, i.e., 6.03%, 36.34%, 59.62%, 64.61%, and 46.15%, respectively. T_l_ at 40 °C, when the molecular weight increased from 23 to 109 k, 201 k, 360 k, 710 k, the form I contents of five samples were 0.78%, 1.48%, 4.12%, 4.67% and 3.88%, respectively. When T_l_ was at −50 °C, the form I contents of five samples were 1.51%, 1.75%, 23.17%, 52.25%, and 20.06%, respectively. It should be mentioned that the glass transition temperature of the poly(1-butene) sample is about −27 °C; when the T_l_ was at −50 °C, the molecular chain mobility in the PB-1 sample was inhibited and cannot form a crystal nucleus, but it can be found that the PB-1 sample still had the melting peak of crystal form I, which means that the transition of crystal form II to I could proceed. Although nuclei cannot be formed when T_l_ was at −50 °C, the internal stress generated during the rapid cooling from 50 to −50 °C still existed, then during the heating process, the sample would pass through the −10 °C region with fast nucleation, so the nucleation should happen rapidly under internal stress. Finally, the crystal form I was also obtained. 

When T_l_ was at −10 °C, the melting peak area of crystal form II was the smallest, the peak of crystal form I was the largest, and the crystal transformation rate of the poly(1-butene) sample was the fastest. The obtained crystal form I had the highest content, and other lower or higher annealing temperatures, such as −50, −20, 0, 10, 40 °C, would reduce the nucleation rate. This was because the molecular chain mobility was limited when the sample was annealed at too low T_l_ (−50, −20 °C), which would lead to the formation of fewer crystal nuclei. Fewer nuclei were also obtained when the samples were annealed at a higher T_l_ (0, 10, 40 °C). This was consistent with the conclusion reached by Men et al., who found that the best T_l_ of the PB-1 sample was at −10 °C [22]. However, compared with the broad molecular weight distribution of PB-1 (*M*_w_ = 711 k) used by Men et al., it can be found that the narrow molecular weight distribution sample F5 (*M*_w_ = 710 k) resulted in more crystalline form I under the same annealing condition. 

As shown in Figure 6b, the M_w_ is 360 k. When T_l_ was at −50, −20, −10, 0, 10, and 40 °C, X_I_ was 52.25%, 57.42%, 64.61%, 56.27%, 47.86%, 4.67%, respectively. X_I_ was the highest among the five samples. While the M_w_ is 23 k, the form I content of sample F1 under different T_l_ was 1.51%, 4.67%, 6.03%, 4.11%, 2.41%, 0.78%, respectively. While the M_w_ is 710 k, the form I content of sample F5 under different T_l_ was 20.06%, 37.97%, 46.15%, 40.55%, 27.24% and 3.88%, respectively. It can be found that under the same T_l_, with increasing the molecular weight, X_I_ obtained by step-by-step annealing firstly showed an increasing trend and then decreased. Previous studies had shown that internal stress can accelerate nucleation [30]. The linked molecular chains between platelets (mainly composed of entangled loops and tie molecules) play an important role in the transfer of internal stress, and the content of linked molecules increases with the molecular weight [31]. When different samples were cooled from isothermal crystallization temperature (50 °C) to T_l_, the supercooling degree was almost the same, and the growing rate of the crystal nucleus was affected by the molecular chain mobility. When the molecular weight of PB-1 increases from 23 k (F1) to 360 k (F4), the number of linked molecules increased with the molecular weight, which can transmit more internal stress during the cooling process and facilitate nucleation, thereby accelerating the progress of nucleation. In addition, the segments of the PB-1 sample, which can be diffused and rearranged, also had mobility, they were beneficial to the nucleus growth, and finally more crystal form I was obtained in the F4 sample. As the molecular weight continued to increase, the number of linked molecules increases, but the molecular chain mobility of high-molecular weight segments was limited and not beneficial to the growth of crystal nuclei. For example, when T_l_ was at −20 °C, the molecular weights from F1 (23 k) to F4 (360 k), the crystal form I increased from 4.67% to 57.42%. However, from F4 (360 k) to F5 (710 k), the crystal form I decreased from 57.42% to 37.97%. Therefore, the content of crystal form I obtained by step-by-step annealing firstly showed an increasing trend and then decreased.

### 3.4. Influence of High Annealing Temperature T_h_ and Molecular Weight on Crystal Transformation

**Figure 7:**
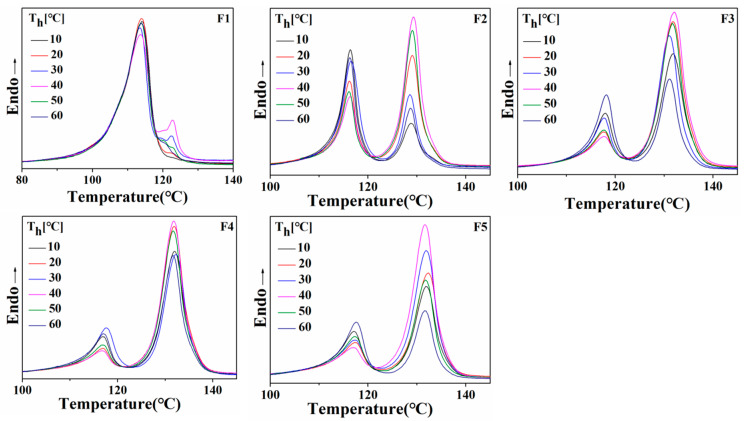
DSC melting curves of five samples annealed at different high annealing temperatures (T_h_).

**Figure 8:**
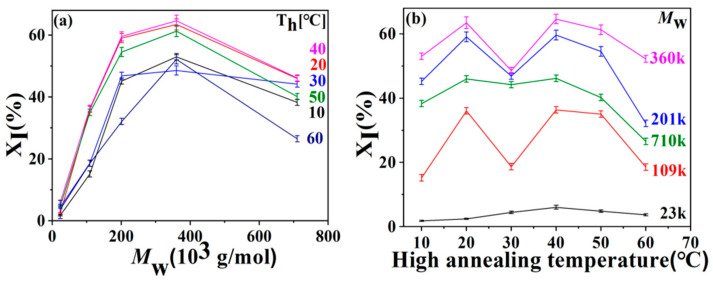
Variation of the form I content (X_I_) of samples with different molecular weights (**a**) or different high annealing temperatures (T_h_) (**b**).

Five samples (F1-F5) were annealed at T_1_= −10 °C for 60 min after isothermal crystallization; then, they were annealed at different high annealing temperatures T_h_ (10, 20, 30, 40, 50, 60 °C) for 220 min and finally heated to 180 °C. The recorded DSC melting curves are shown in Figure 7. X_I_ data are listed in Table S3. The plots of X_I_ versus *M*_w_ or T_h_ of the five samples are shown in Figure 8. 

While the *M*_w_ is 360 k, the contents of crystal form I from 10 to 60 °C were 53.00%, 63.44%, 48.57%, 64.61%, 61.26% and 52.18%, respectively. While the *M*_w_ is 23 k, the contents of crystal form I at a different high annealing temperature T_h_ were 1.77%, 2.43%, 4.43%, 6.03%, 4.81% and 3.69%, respectively. While the *M*_w_ is 710 k, the contents of crystal form I at different T_h_ were 38.31%, 45.97%, 44.17%, 46.15%, 40.14%, 26.51%, respectively. It can be seen that when the T_h_ was at 40 °C, the content of crystal form I obtained among the five samples was the most. The transition speed of the crystal form II was the fastest at 40 °C, while it slowed down when the temperature was lower or higher than 40 °C. This was inconsistent with the previous study, which suggested that the crystal transformation was fastest at room temperature, because the nucleation rate was the fastest at a lower temperature and the growth rate was the fastest at a higher temperature; therefore, the best annealed temperature must be optimized to satisfy a larger nucleation and growth rate. During the step-by-step annealing process, the crystal nucleation had already formed, and it only needed to satisfy its faster growth rate at a higher temperature. With the increase in annealing temperature T_h_, the molecular chain mobility was enhanced. When the T_h_ was too high, the crystals obtained by nucleation cannot stably exist. 

As shown in Figure 8, when T_h_ was at 10 °C, the crystal form I contents of samples were 1.77%, 15.17%, 45.18%, 53.00% and 38.31%, respectively. When T_h_ was at 40 °C, they were 6.03%, 36.34%, 59.62%, 64.61%, and 46.15%, respectively. When T_h_ was at 60°C, they were 3.69%, 18.54%, 32.10%, 52.18% and 26.51%, respectively. At the same T_h_, with increasing molecular weight, X_I_ firstly showed an increasing trend and then decreased by the step-by-step annealing process. For the F4 (360 k) sample, the content of crystal form I was the highest in five samples, and it was 53.00%, 63.44%, 48.57%, 64.61%, 61.26% and 52.18%, respectively, corresponding to T_h_ at 10, 20, 30, 40, 50, and 60 °C. With the increase in molecular weight, the driving force for nucleation increased, which made it easier to overcome the energy barrier and had the advantage of nucleation; the molecular chain ability of the PB-1 sample was not inhibited, which has the advantage of crystal growth, and finally more crystal form I was obtained. As the molecular weight increased to a high level, the molecular chain ability of the PB-1 sample was more and more restricted, which was unbeneficial to the growth of the crystal nuclei. Therefore, X_I_ firstly showed an increasing trend and then decreased with the increasing molecular weight. It should be mentioned that this was inconsistent with the viewpoint of Winter et al., who observed that the speed of crystallization was independent of molecular weight [28]. It is possible that the reason for this is that the used samples (iPB398, iPB295, iPB177 and iPB116) had a broad molecular weight distribution (As shown in Table 1, PDI from 3.1 to 4.6), which may have affected the final experimental results.

### 3.5. Influence of Low-Temperature Annealing Time t_l_ and Molecular Weight on Crystal Transformation

**Figure 9:**
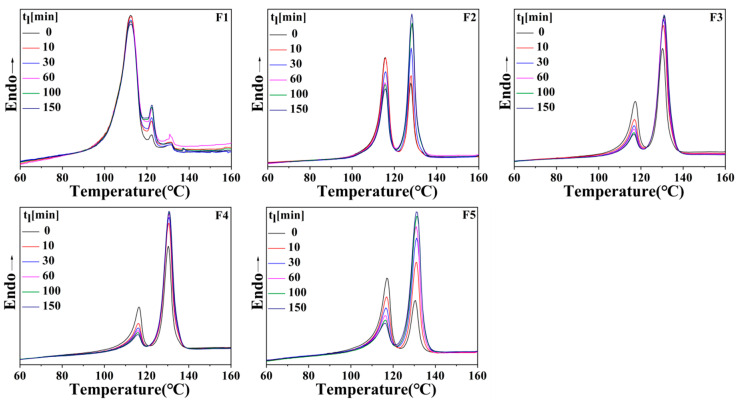
DSC melting curves of five samples annealed at different low-temperature annealing times (t_l_).

**Figure 10:**
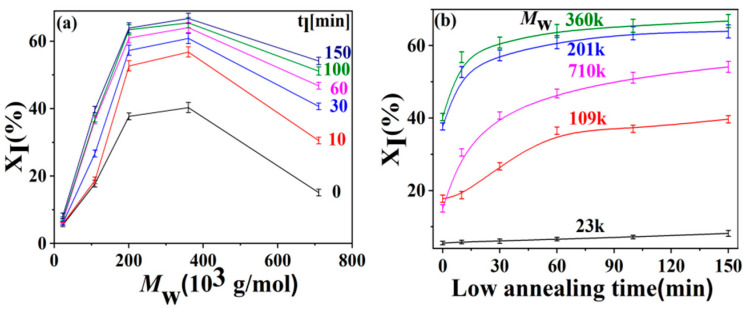
Variation of the form I content (X_I_) of samples with different molecular weights (**a**) or low-temperature annealing times (t_l_) (**b**).

Five samples (F1-F5) were annealed at −10 °C (T_l_) with different low-temperature annealing times t_l_ (0, 10, 30, 60, 100, 150 min), then annealed at 40 °C (T_h_) for 220 min(t_h_). Figure 9 recorded the DSC curves of PB-1 samples after heat treatment. X_I_ are listed in Table S4. Figure 10 shows the plot of X_I_ versus the *M*_w_ or t_1_. 

When the *M*_w_ is 360 k, X_I_ at the different annealing times t_l_ (0, 10, 30, 60, 100, 150 min) was 40.32%, 56.82%, 60.85%, 64.06%, 65.49%, and 66.79%, respectively. When *M*_w_ is 23 k, it was 5.50%, 5.81%, 6.05%, 6.60%, 7.19% and 8.19%, respectively. When *M*_w_ is at 710 k, it was 15.08%, 30.54%, 40.69%, 46.79%, 51.16% and 54.16%, respectively. X_I_ was the most in the five samples when M_w_ is 360 k. As in the previous study, we also observed that crystal form I content firstly increased with annealing time t_l_, and then the transformation rate slowed down with time and gradually reached a plateau. However, it was obtained that the time to reach plateau was different. The transformation rate in the low molecular weight F1 (23 k) was relatively slower, and increased linearly with the annealing time t_l_. When molecular weights are 109 and 710 k, it can be seen that the plateau was almost reached when the t_l_ was 120 min. While the M_w_ of samples are 201 and 360 k, it took less time (t_l_ = 60 min) to reach the plateau. The internal stress in the cooling process would relax slowly with the time, and the resulting promotion effect on the crystal transition would gradually decrease. Therefore, the form I increased rapidly with t_l_ at the beginning, and then the rate slowed down with time and gradually reached a plateau.

### 3.6. Influence of High-Temperature Annealing Time t_h_ and Molecular Weight on Crystal Transformation

**Figure 11:**
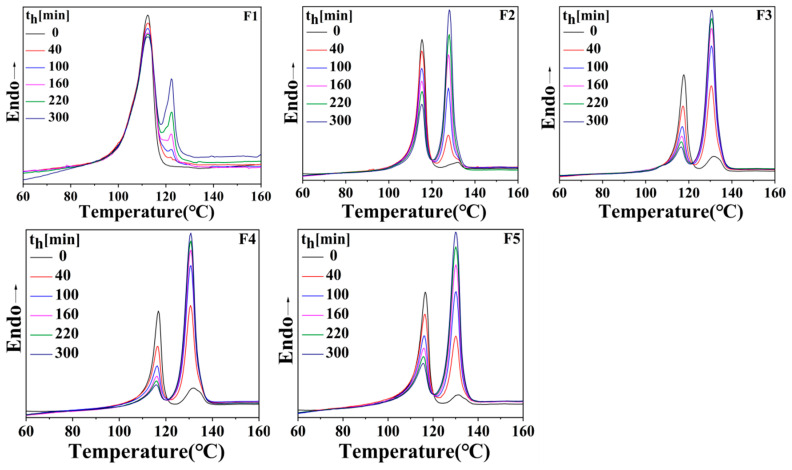
DSC melting curves of five samples annealed at different high-temperature annealing times (t_h_).

**Figure 12:**
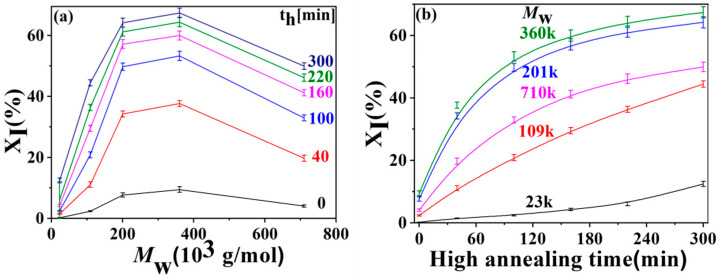
Variation of the form I content (X_I_) of samples with different molecular weights (**a**) or high-temperature annealing times (t_h_) (**b**).

Finally, five samples (F1-F5) were annealed at T_1_= −10 °C for 60 min, then annealed at 40 °C (T_h_) for different time (t_h_ =0, 40, 100, 160, 220, or 300 min). Figure 11 recorded the DSC curves of PB-1 samples after heat treatment. X_I_ are listed in Table S5. Figure 12 shows the plot of X_I_ versus the *M*_w_ or t_h_. 

As shown in Table S5, when t_h_ was at 0 min, the contents of the F1 (23 k)-F5 (710 k) crystal form I were 0.21%, 2.35%, 7.62%, 9.44%, and 4.04%, respectively. When t_h_ was at 100 min, they were 2.41%, 20.84%, 49.77%, 53.30%, and 32.96%, respectively, and they were 12.43%, 44.45%, 64.19%, 67.40%, and 49.93%, respectively, when T_h_ was at 300 min. Firstly, the same conclusion can be obtained as mentioned above, namely, when *M*_w_ is 360 k, the content of poly(1-butene) crystal form I was the highest in all. Then, it can be observed that the crystal form I content increased with the high annealing time t_h_ in Figure 12. When the molecular weights of the samples were relatively lower (F1 and F2), X_I_ monotonously increased with high-temperature annealing time t_h_. However, when the molecular weights were higher (F3, F4 and F5), PB-1 crystal form I increased rapidly with t_h_ at the beginning, and the transformation speed was also faster at this time, then transformation speed slowed down with time.

## 4. Conclusions

In this study, the annealing temperature and time were external factors and molecular weight was an internal factor. How to influence the crystal transformation from crystal form II to form I for PB-1 samples was researched in detail by means of the DSC technique. Specifically, in order to avoid the interference of molecular weight distribution on crystallization behavior, five narrow distribution PB-1 samples with different molecular weights (from 23 to 710 k) were selected. The annealing procedure was divided into two steps, i.e., nucleation and growth, and the optimal nucleation and growth temperatures were separately investigated. The temperature and time dependence of samples were also explored. Samples were annealed stepwise at different T_l_/t_l_ or T_h_/t_h_, or they were annealed at one temperature for the same time. The results can be summarized as follows.

(1) When annealed at one temperature, X_I_ obtained by annealing at −10 °C was more than that obtained by annealing at 40 °C. Compared with the single annealing temperature, the step-by-step annealing process can significantly speed up the transformation rate from crystal form II to form I.
(2) PB-1 samples with different molecular weights have the same dependence on annealing temperature, and the optimal low annealing temperature T_l_ and high annealing temperature T_h_ were −10 °C and 40 °C, respectively. The crystal form I obtained by step-by-step annealing at these two temperatures had the highest content and the fastest transformation rate.
(3) Under the same annealing temperature T_l_ or T_h_, with the increasing of molecular weight, X_I_ obtained by the step-by-step annealing process firstly showed an increasing trend and then decreased. The molecular weight of sample F4 (360 K) was neither lowest nor highest in five samples, but its form I content was the highest one in all, which was because that molecular weight had double influence on the polymorphic transition from form II to form I.
(4) Under the same annealing temperature T_l_ and T_h_, X_I_ firstly increased with annealing time t_l_, then the rate slowed down and gradually reached a plateau, but the time to reach the plateau was different due to the different molecular weights of the five samples. X_I_ of the samples with a relatively lower molecular weight monotonously increased with annealing time t_h_. While the molecular weight was higher, X_I_ increased rapidly with t_h_ at the beginning, and then transition speed slowed down.

In conclusion, the research provides an effective method for accelerating or adjusting the transformation of poly(1-butene) from crystal form II to form I.

## Figures and Tables

**Table 1:** Sample information and annealing conditions in literatures.

Sample	Sample Type	*M*_w_ (kg/mol)	PDI	Annealing Method	Manufacturer	References
DP8510M	Copolymer	153	2.2	Stepwise Annealing	Lyondell Basell Industry	Men et al., 2021 [6]
PB0400M	Homopolymer	188	2.7	Single temperature annealing	Lyondell Basell Industry	Sun et al., 2020 [12]
PB0400M	Homopolymer	188	2.7	Stepwise Annealing	Lyondell Basell Industry	Men et al., 2021 [6] Sun et al., 2019 [13] Men et al., 2017 [24]
PB0110 PB8640 PB8220 PB8510	Homopolymer Copolymer Copolymer Copolymer	742 552 414 203	3.3 3.5 2.7 2.6	Single temperature annealing/ Stepwise Annealing	Lyondell Basell Industry	Chen et al., 2018 [14]
iPB	Homopolymer	548	3.9	Single temperature annealing	Shandong Dongfang Hongye Chemical Co., Ltd. China	He et al., 2020 [15]
PB8340M	Copolymer	28.1	4.3	Stepwise Annealing	Lyondell Basell Industry	Wang et al., 2018 [16]
PB-L	Homopolymer	8.0	2.7	Stepwise Annealing	Tianjin University	Men et al., 2021 [23]
PB0800	Homopolymer	77	3.0	Stepwise Annealing	Lyondell Basell Industry	Men et al., 2017 [24]
F2 F4 F6 F8 F10 F12	Homopolymer	31.9 85.3 135.1 239.7 710.0 1029.8	1.53 1.10 1.09 1.14 1.44 1.40	Single temperature annealing	Changchun Institute of Applied Chemistry	Xue et al., 2020 [26]
iPB398 iPB295 iPB177 iPB116	Homopolymer	398 295 177 116	3.8 4.6 3.3 3.1	Single temperature annealing	Shell	Winter et al., 2002 [28]

**Table 2:** Molecular weight and molecular weight distribution of five samples.

Sample	*M*_w_ (10^3^ g/mol)	PDI
F1	23	1.09
F2	109	1.10
F3	201	1.10
F4	360	1.20
F5	710	1.44

**Table 3:** DSC data of five samples.

Sample	T_m_ (°C)	∆H_m_ (J/g)	T_c_ (°C)	∆H_c_ (J/g)
F1	113.8	44.2	82.4	42.4
F2	116.9	43.6	88.4	43.1
F3	118.8/132.6	36.8/0.2	87.4	36.6
F4	117.9/132.2	35.4/0.2	88.1	34.1
F5	117.9/132.5	33.3/0.1	84.7	32.7

## Data Availability

The data presented in this study are available on request from the corresponding author.

## Supplementary Materials

The following are available online at https://www.mdpi.com/article/10.3390/polym15040800/s1, Table S1: The crystal Form I contents of five samples at different stepwise annealing condition, Table S2: The crystal Form I contents of five samples at different low annealing temperatures(T_l_), Table S3: The crystal Form I contents of five samples at different high annealing temperatures (T_h_), Table S4: The crystal Form I contents of five samples at different low annealing time (t_l_), Table S5: The crystal Form I contents of five samples at different high annealing time (t_h_).
