# Supplementary material for: Effect of Annealing Process and Molecular Weight on the Polymorphic Transformation from Form II to Form I of Poly(1-butene)
Source: Polymers (Basel). 2023 Feb 5;15(4):800. doi: 10.3390/polym15040800 (PMC9965630; doi:10.3390/polym15040800)
Supplement: Supplementary file 1 [file polymers-15-00800-s001.zip › polymers-2148040-SM.pdf]

polymers

MDPI

Supporting Information

Effect of Annealing Process and Molecular Weight on the Polymorphic Transformation from Form II to Form I of Poly(1-Butene)

Zhenkang Zhang [1,2], Yanhu Xue [1,*], Rui Li [2], Wei Liu [2], Peng Liu [1] and Xiangling Ji [2,*]

[1] College of Chemical Engineering and Materials Science, Tianjin University of Science and Technology, Tianjin 300457, China
[2] State Key Laboratory of Polymer Physics and Chemistry, Changchun Institute of Applied Chemistry, Chinese Academy of Sciences, Changchun 130022, China
* Correspondence: xueyh@tust.edu.cn (Y.X.); xlji@ciac.ac.cn (X.J.); Tel.: +86-22-60601140 (Y.X.); +86-431-85262876 (X.J.)

Table S1. The crystal Form I contents of five samples at different stepwise annealing condition.

Sample	M_w(10^3 g/mol)	$X_{I,\ T=-10℃}$(%)	$X_{I,\ Tl=-10℃,\ Th=40℃}$ (%)	$X_{I,\ Th=40℃}$ (%)
F1	23	1.02	6.03	0.78
F2	109	5.28	36.34	1.48
F3	201	15.23	59.62	4.12
F4	360	19.14	64.61	4.67
F5	710	18.66	46.15	3.88

Table S2. The crystal Form I contents of five samples at different low annealing temperatures (T_l).

Temperature	X_I (%)				
(°C)	23k	109k	201k	360k	710k
T_l= −50	1.51	1.75	23.17	52.25	20.06
T_l= −20	4.67	5.46	38.88	57.42	37.97
T_l= −10	6.03	36.34	59.62	64.61	46.15
T_l=0	4.11	12.15	33.87	56.27	40.55
T_l=10	2.41	12.03	20.61	47.86	27.24
T_l=40	0.78	1.48	4.12	4.67	3.88

Table S3. The crystal Form I contents of five samples at different high annealing temperatures (T_h)

Temperature (°C)	X_I (%)				
	23k	109k	201k	360k	710k
T_h=10	1.77	15.17	45.18	53.00	38.31
T_h=20	2.43	36.03	59.06	63.44	45.97
T_h=30	4.43	18.62	46.85	48. 57	44.17
T_h=40	6.03	36.34	59.62	64.61	46.15
T_h=50	4.81	35.02	54.52	61.26	40.14
T_h=60	3.69	18.54	32.10	52.18	26.51

Table S4. The crystal Form I contents of five samples at different low annealing time (t_l)

Time(min)	X_I (%)				
	23k	109k	201k	360k	710k
t_l= 0	5.50	17.75	37.70	40.32	15.08
t_l= 10	5.81	18.73	52.71	56.82	30.54
t_l= 30	6.05	26.68	57.32	60.85	40.69
t_l= 60	6.60	36.53	60.98	64.06	46.79
t_l= 100	7.19	37.07	63.41	65.49	51.16
t_l= 150	8.19	39.70	63.96	66.79	54.16

Table S5. The crystal Form I contents of five samples at different high annealing time (t_h)

Time(min)	X_I (%)				
	23k	109k	201k	360k	710k
t_h= 0	0.21	2.35	7.62	9.44	4.04
t_h= 40	1.45	11.08	34.25	37.66	19.71
t_h= 100	2.41	20.84	49.77	53.30	32.96
t_h= 160	4.24	29.51	57.17	59.97	41.21
t_h= 220	6.07	36.34	61.21	64.33	46.15
t_h= 300	12.43	44.45	64.19	67.40	49.93
